# Diagnosing rare diseases and mental well-being: a family’s story

**DOI:** 10.1186/s13023-023-02648-y

**Published:** 2023-03-06

**Authors:** Zheqing Zhang

**Affiliations:** Medical Sciences Division, University of Oxford, Oxford, UK

**Keywords:** Rare disease, Vascular Ehlers-Danlos syndrome, Patient perspective, Mental well-being, Diagnosis

## Abstract

When we experience symptoms, most of us walk into the clinic or hospital expecting immediate answers. For individuals with a rare condition, the path to diagnosis can be tortuous, involving months to years of waiting and a seemingly interminable search for answers. All this while, physical and psychological stress can negatively impact mental health. Each diagnostic journey is unique, but they epitomise common themes and inadequacies of the medical system. This article presents the stories of two sisters whose diagnostic journeys diverged then converged, reflecting on the impact of these experiences on mental well-being and what we can learn going forward. Hopefully, with more research and knowledge, we can catch these conditions earlier and provide better recommendations for treatment, management and prevention.

## Introduction

There are many things that outsiders fail to appreciate about the lives of patients with rare diseases, which each affect less than 1 in 2,000 people in Europe [1]. Different national definitions of ‘rare disease’ exist, ranging from prevalence of 5 to 80 per 100,000 [2]. According to a 2019 analysis, rare diseases affect a conservative estimate of 3.5% to 5.9% of the global population, equivalent to 263 to 446 million individuals worldwide [2]. A 2018 report provided a similar estimate of 350 to 400 million, or 3.5 million in the UK, meaning that about 1 in 17 people will have a rare disease at some point in their lives [3]. Therefore, while rare diseases are individually rare, they collectively represent a substantial burden. Between 2008 and 2018, rare disease patients in the process of diagnosis cost NHS England more than £3.4 billion [3], highlighting unresolved challenges and inefficiencies. For rare disease patients, the diagnostic delay can range from months to decades, averaging four to five years [4]. On average, rare disease patients receive three misdiagnoses and consult with five doctors before receiving an accurate diagnosis [5, 6].

The long diagnostic odyssey, with some patients remaining undiagnosed or misdiagnosed, can be mentally and emotionally draining for patients and their loved ones. Based on responses from the 2019–2020 H-Care Survey, the EURORDIS Rare Barometer Survey report revealed that individuals with rare diseases give their healthcare experience a medium–low rating, with a lower average score than those with chronic diseases [7]. A survey of 1231 patients and 564 carers in the UK found that due to their rare condition, 90% or more of participants had felt worried or anxious, stressed, emotional, low or depressed, and/or angry or frustrated at least some of the time, with experience of suicidal thoughts reported by 36% of patients and 19% of carers [8]. However, nearly half of those surveyed had never been asked about mental health by healthcare professionals [8], highlighting a gaping disparity between patient needs and healthcare provision. Prioritisation of patients’ mental well-being in the process of diagnosis and treatment should be at the forefront of our collective effort in the fight against rare diseases, bringing medical and healthcare professionals closer to the exigencies of patients and their families. This article will compare and contrast the diagnostic journeys of two sisters as a window into the patient perspective, reflecting on the impact of these experiences on their mental well-being and the compelling lessons they leave for all of us.

### Case study

It took the death of two siblings for Sarah to be diagnosed. In March 2000, Sarah and Lizzie were three and seven years old, respectively, when their older brother died suddenly from an aortic dissection, aged fifteen. There were no warning signs; he started throwing up, fainted, and was rushed to the hospital, but it all ended in less than twelve hours. Lizzie was born with a heart murmur and had balloon valvuloplasty at age seven for pulmonary stenosis. No other family members had cardiovascular issues, which led doctors to believe that her brother had a de novo mutation causing Marfan syndrome. The rest of the family tested negative for the condition, and the case was closed.

Eighteen years later, in August 2018, Sarah began experiencing a series of unexplained symptoms, most of which were neurological and pulmonary in nature. In March 2019, she suffered a spontaneous pneumothorax. However, multiple examinations ruled out infection, cancer, and lupus. “My brother died from an aortic dissection and I think this is something related.” Sarah remembers repeating this to her doctors, but they never referred her to a geneticist. Sarah suffered two further lung collapses in June 2019, underwent surgery, and had yet another lung collapse six months later. Doctors suspected a connective tissue disorder, but no one considered vascular disorders or genetic testing.

In March 2020, 27-year-old Lizzie presented to the hospital at 39 weeks of gestation with abdominal pain and vaginal bleeding. She was diagnosed with HELLP syndrome, admitted for emergency caesarean delivery, and developed unexplained placental abruption and liver laceration [9]. Three days later, further respiratory and cardiovascular complications ensued, and doctors failed to save her despite their best efforts. Autopsy revealed an aortic rupture, and genetic testing was consistent with vascular Ehlers-Danlos syndrome (VEDS), caused by a mutation in the *COL3A1* gene. In the remaining family, Sarah was the only person to test positive, and it was assumed that this condition had also afflicted their brother.

Ehlers-Danlos syndromes (EDS) are a family of rare inherited connective tissue disorders. Vascular EDS, the most severe form, is characterised by fragile arteries, muscles and internal organs, usually caused by a mutation in the *COL3A1* gene [10]. Looking back, Sarah and Lizzie exhibited subtle signs such as easy bruising and thin skin. However, in most cases, outward features are not obvious until a major catastrophic event occurs. As VEDS has autosomal dominant inheritance, parents with affected children would usually show symptoms [11]. This was not so for Sarah’s family; instead, Sarah’s mother was not phenotypically affected but likely had mosaicism, meaning that only some of her cells harbour a genetic mutation, which was passed on to three of her eight children.

Sadly for Lizzie, her post-mortem diagnosis came too late, and her loved ones were left to deal with the anguish. This was especially harrowing for her husband, Todd, and their baby girl, Juniper, as the celebration of a new life was marred by a tragic death. Simultaneously, these experiences took a toll on the mental well-being of Sarah and her family. The diagnosis brought clarity and healing, as pieces of the puzzle finally fit together. Thankfully, genetic testing revealed that Juniper had been spared. However, as things happened so quickly, Sarah felt she never had enough time to fully process her emotions. Her hospital visits coincided with her senior year of college, adding stress onto an already difficult degree. Shortly after graduation, her life was turned upside down by her sister’s tragedy, against the backdrop of a global pandemic.

For Sarah, grappling with the grief of losing her sister and the lifelong implications of her own diagnosis was a mental whirlwind—a strange mix of relief, guilt, anger, shock, and confusion. Arguably, the premature deaths of her siblings were worse outcomes, but they were spared the psychological trauma and survivor’s guilt, which affected Sarah profoundly. It bothered her that she had not found an answer for an entire year, when continuously seeking second opinions left her exhausted and bewildered. She was angry at herself for not pursuing it more, and angry at her doctors for not diagnosing it earlier. “I can’t believe we had to lose another person to finally get an answer,” she laments [12]. Sarah must now accept that none of this was her fault. Retrospectively, many clues were missed, and Lizzie’s doctors should have been informed of her sister’s pulmonary problems. More efficient communication between healthcare providers in different locations could have helped. Additionally, this underlines the importance of taking a family history, and maintaining curiosity and vigilance.

The full gravity of the situation dawned on Sarah much later, when she began worrying about her life, her future, and the possibility of her family losing her too. Having witnessed two deaths, she was acutely aware of her mortality. Her anxiety and ADHD-like symptoms were exacerbated by numerous life-threatening events and fear that something could happen out of the blue. Chronic stress makes her weary, and she gets nervous when travelling or away from her doctors. Mental health issues are common alongside rare diseases, and psychological support, both professional and otherwise, must not be neglected [8]. Luckily, Sarah has assembled her own coordinated care team, which she calls her “Dream Team”, including a cardiologist, pulmonologist, neurologist, psychotherapist, and nutritionist, and also benefits from massage therapy and acupuncture. Through communities like The VEDS Movement, she has found friends with the same condition, adding to her support network. In many ways, her diagnosis was a turning point that pushed her to take charge of her treatment and management.

As patients heal from emotional pain, their diagnosis can provide a clearer direction for the future and empower them to make a difference. Newfound knowledge becomes a catalyst for positive change, as the focus shifts from “What is wrong with me?” to “How can I help others?” Sarah is grateful to have found answers after only a year of debilitating symptoms, and finds solace in knowing that her sister’s death probably saved her life. When we met in November 2022, more than two years post-diagnosis, I was astounded by her optimism. Like many, she has become a staunch patient advocate. Sharing her story is her way of educating the public and medical community, in the hopes that others will avoid such terrible ordeals on the path to diagnosis. Lizzie’s legacy persists through Sarah and Todd, who share her story in interviews and podcasts. Discussing Lizzie’s case, a doctor proposed integrating rare diseases into the medical school curriculum [13]. As many physicians are unfamiliar with rare diseases, patients often find themselves the expert. This defies tradition, recognising the value of doctors learning from patients. For instance, Sarah’s pulmonologist went on to educate himself and his colleagues on VEDS. Healthcare providers should constantly identify and fill gaps in knowledge, never forgetting that medicine, especially timely diagnosis, requires lifelong learning.

When standard medical examinations detect nothing overtly wrong, rare disease symptoms can be dismissed as “purely psychological” or “nothing major”. Once, Sarah saw a doctor who told her “I think you just have anxiety”, without perusing her medical charts. For all three siblings, nothing seemed amiss, then things deteriorated rapidly. Many VEDS patients are only identified after a catastrophic or fatal event, and it is likely that many with mild phenotypes remain undiagnosed [11]. How we can improve testing and detection of VEDS and other rare diseases remains an open question.

Compared to her siblings, Sarah may have been “the lucky one”, but the story does not end here. Ripples of mental distress extend beyond the diagnosis and patients themselves. For Todd and Juniper, the loss of wife and mother will haunt them for decades. For Sarah, she has to navigate her adult life with courage and faith. For all of us, a pressing question remains: How can we improve the physical and mental wellbeing of patients before (and after) diagnosis?

## Discussion

This case study represents a mere snapshot of a much wider problem. Of the approximately 30 million individuals living with a rare disease in Europe, 50% are undiagnosed [14]. In a 2015 UK-based survey, 87% of undiagnosed patients felt they did not have enough information and support during the diagnostic process [5]. Lack of diagnosis provides a barrier to accessing treatment and care. Moving forward, a cogent lesson is to improve medical education and research, ensuring that doctors in various specialties are cognizant of rare disease presentations.

The UK Rare Diseases Framework 2021 and the England Rare Diseases Action Plan 2022 signal political attention and are steps in the right direction. Still, it is important that such actions are brought to the community level. Diagnosis could be accelerated by expanding the repertoire of preconception screening, newborn screening, and early genetic testing, especially with advancements in next-generation sequencing and related technologies. A research pilot, the Newborn Genomes Programme, is using whole-genome sequencing to screen for rare genetic conditions in newborns [15], in addition to the routine blood spot (heel prick) test. Digital tools can provide quick access to information, as well as virtual consultations with multiple specialists at once, especially since rare disease patients tend to present with symptoms affecting different body systems. A multidisciplinary team may benefit rare disease patients, even on the road to diagnosis. Coordinated care is vital to simultaneously address physical and mental repercussions of disease. Besides, collaboration and communication are critical at various levels, including between healthcare professionals, hospitals, and research groups. Streamlining access to patient data across healthcare providers can enable earlier recognition of subtle signs. An example of higher-level collaboration is Solve-RD (Solving the Unsolved Rare Diseases), a research project involving hundreds of rare disease experts across Europe [14], enabling analysis of larger datasets to create a “genetic knowledge web” [16]. Hopefully, this and similar approaches will continue to unveil new diagnostic tools for rare conditions.

## Final remarks

While this article focussed on the diagnostic odyssey, the journey does not end here. Improvements in treatment, education, and research must naturally follow. A report published by Rare Disease UK in 2019 put forth several recommendations, revolving around improving diagnosis and early intervention; modernising rare disease care and treatments; enabling access to accurate information; and protecting and enhancing rare disease research [6]. A 2021 EURORDIS report identified three priorities to enhance the healthcare experience: follow-up consultations, more signposting on resources and support, and psychological assistance [7]. Communication between patients, clinicians, and researchers needs to improve, perhaps through a more streamlined process written into hospital protocols, alongside a more holistic and personalised approach with both the mental and physical well-being of the patient at its core.

## Data Availability

See References.

## Abbreviations

NHS: National health service
HELLP: Haemolysis, elevated liver enzymes, low platelet count
VEDS: Vascular Ehlers-Danlos syndrome
ADHD: Attention deficit hyperactivity disorder
